# Awareness of Occupational Hazards and Utilization of Safety Measures among Welders in Aksum and Adwa Towns, Tigray Region, Ethiopia, 2013

**DOI:** 10.1155/2019/4174085

**Published:** 2019-01-21

**Authors:** Berhe Beyene Gebrezgiabher, Desalegn Tetemke, Tesfaye Yetum

**Affiliations:** ^1^Department of Public Health, College of Health Science, Aksum University, Axum, Ethiopia; ^2^Federal Ministry of Health, Consultancy Service, Addis Ababa, Ethiopia

## Abstract

**Introduction:**

At present, it is estimated that more than one million workers are employed as welders worldwide with more than three million performing welding as part of their work duties.

**Objectives:**

The aim of this study was to assess workers' level of awareness towards occupational hazards and their adherence to safety measures.

**Methodology:**

An institution-based cross-sectional study was conducted from February 25 to March 10, 2013 among welding factory workers. The study included 278 workers selected by simple random sampling, and data were collected by using structured and pretested questionnaire. The data were entered and analyzed using SPSS version 16 statistical package. Logistic regression analysis was carried out to found the effect of the independent variables on the dependent variables.

**Result:**

One hundred thirty-five (51.9%) respondents had knowledge on occupational hazards and 225 (86.5%) workers used personal protective equipments. Variables such as work experience, work type, safety training, work regulation, and guideline had significant association with the knowledge of respondents: (AOR: 0.44 (0.19, 0.99)), (AOR: 0.38 (0.22, 0.65)), (AOR: 0.33 (0.17, 0.63)), and (AOR: 0.31 (0.15, 0.67)), respectively. Educational status, work experience, safety training, and availability of work regulation were found to be associated with PPE use (AOR: 13.20 (10.65, 16.46)), (AOR: 0.03 (0.003, 0.34)), (AOR: 0.02 (0.01, 0.09)), and (AOR: 0.06 (0.02, 0.21)), respectively.

**Conclusion and Recommendation:**

Nearly half of respondents had knowledge about occupational hazards and high proportion of study subjects were used personal protective equipments. Safety and health training was the common factor to increase knowledge and personal protective usage practicing habits. Employers and other responsible bodies should encourage training and regular supervision should be made including on workers' safety and well-being.

## 1. Introduction

The World Health Organization (WHO) considers the workplace a priority setting for health promotion in the 21st century. Like other settings where WHO has developed health-promoting initiatives (schools, cities, hospitals, and industries), the workplace can have a very positive impact on the health and well-being of workers, their families, communities, and society at large [1].

Safe work and workplace, for increased production and higher productivity, are necessary and hence promotion and protection of safe work and workplace are the complementary aspects of industrial development [2, 3]. However, industrial occupations may create unsafe work and work environment because of the inherent sources of hazard present in their material, process, technologies, or products. These sources of hazards may pose the risk of accidents and work related disease to the people within the industrial premises in particular and the general public in the vicinity and the environment in general [4].

Welding is the process in which metal or other thermoplastic materials are joined together by the application of heat or pressure, or both with or without the use of filler metal [5].

At present, it is estimated that more than one million workers are employed as welders worldwide with more than three million performing welding intermittently as part of their work duties. One of the jobs that contribute to these occupational injuries is nonindustrial welding, especially in developing countries including Ethiopia. Welders cut and join metal parts using flame, electric arc, or other sources of heat [6]. The current rapid economic development has brought changes in workplaces in developing countries, including Ethiopia. The organization of occupational health and safety services is not yet resilient enough to handle the growing demands for workers' health in the context of industrialization including for welding workers. A review report conducted in Ethiopia indicates that there are gaps on research, capacity, policy and regulation, training, organizational structure, monitoring, and evaluation as well as database for intervention [7]. Health- and safety-related problems in developing countries have always been there. Welding workers are among the most neglected groups of workers suffering from work-related problems. Most of the evidence published is collected from the developed world, and limited research studies have been conducted so far but not yet in the region regarding welding and health-related problems. Hence, the aim of this study was to assess workers' level of awareness towards occupational hazards and their adherence to safety measures as basic for intervention and problem solving modalities.

## 2. Methods and Materials

A cross-sectional study design using the quantitative method was used. The study was conducted from February 25 to March 10, 2013 among welding factory workers in Axum and Adwa towns. Axum town is found in the central zone of Tigray regional state and is located 1090 km North of Addis Ababa. Similarly, Adwa is also found nearest to Axum 18 km away to eastern direction. All workers engaged in welding (metal and wood work) were the source and study population.

The sample size was determined using a single-proportion formula [8] by using 77.9% knowledge of workers on occupational hazards [9], 95% confidence interval, 5% margin of error, and 10% nonresponse rate. Accordingly, 278 study participants were included for the study. Participants were selected from each working environment proportionally, the frame was prepared from both towns, and study subjects were selected using simple random sampling technique for interviewing.

A pretested and structured questionnaire taken from the literature studies was contextualized (mainly focusing on sociodemographic, knowledge-based and practice on protective measures) and was used to collect the required information. The questionnaire was first developed in English and then translated in to Tigrigna and back to English to check its consistency. Four data collectors who are environmental health professionals and two supervisors were participated to conduct the interview. A day-to-day supervision was made during the whole period of data collection. Training was given for data collectors and supervisors. The principal investigators and supervisors made day-to-day visit during the study, supervised data collection, and handled the questionnaires.

The data were entered, cleaned, edited, and analyzed using SPSS version 16 statistical package. Data cleaning was performed to check for accuracy and consistencies and missed values and variables. The cleaned and edited data were ready for appropriate statistical analysis. The mean, standard deviation, odds ratio, and proportion of the variables were done, and logistic regression analysis was used to measure how the outcome variables (knowledge and safe practice of workers) depend on the covariate variables (working environment and sociodemographic variables).

The result of the analysis was presented using tables, charts, and graphs. Ethical clearance was obtained from the Ethical Review Board of Aksum University, and a supporting letter was obtained from each administrative office of the towns. The purpose and importance of the study were explained to the participants. Data were collected after full informed verbal consent was obtained, and confidentiality of the information was maintained by omitting their names and personal identification or privacy.

## 3. Results

### 3.1. Sociodemographic Characteristics of Respondents

A total of 260 respondents, with (96.3%) response rate, were interviewed. All participants were male. The median age of respondents was 25, and the mean (SD) age of respondents was 24.67 ± (4.9) with approximately equally distributed within the age category. Most of the respondents were between grade nine and twelve or going to secondary school 183 (70.4%) followed by grade one and eight going to primary school 43 (16.5%) in their educational status. Half of the respondents were working in metal work with more than 97% of doing nonwelding-related profession (Table 1).

### 3.2. Knowledge of Study Participants on Occupational Hazards

Slightly more than half of the respondents, 135 (51.9%), heard about occupational hazards and two-thirds of the interviewed respondents knew about fire hazards. Majority, 202 (77.7%), of study subjects had knowledge about electrical hazards. For most of the knowledge questions, respondents had approximately equal proportion for knowing and not knowing them. Most of the respondents, 220 (84.6%), had knowledge of accident prevention.

The mean score of respondents to the knowledge questions out of ten questions was 4 (the mean score was calculated as the total score obtained from the respondents divided by sample size). Respondents whose score 4 and above, 115 (44.2%), were considered knowledgeable on occupational hazards (Table 2).

**Figure 1:**
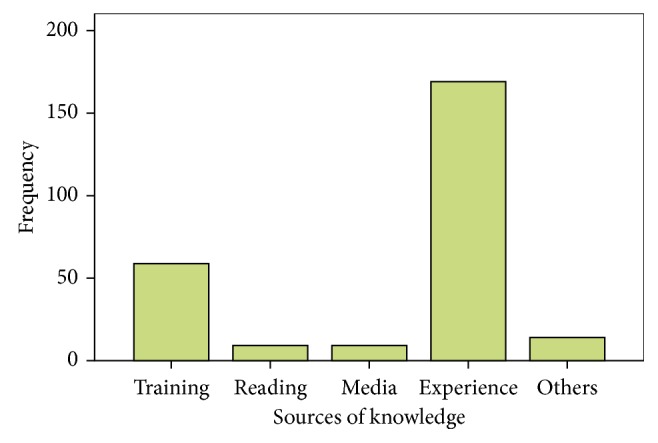
Sources of information of respondents on their knowledge on occupational hazards, among Axum and Adwa welding workers, September 2013.

Among the total study subjects, 62 (23.8%) took training by different bodies, by safety officer, other health professionals and experienced workers 17 (6.5%), 21 (8.1%), and 26 (10%), respectively. Among the total number of respondents, 169 (65%) got their knowledge from experienced workers, 59 (22.7%) acquired from training given their working areas, and 9 (3.5%) for each from media (TV and radio) and from reading (Figure 1).

### 3.3. Safety Practices (PPEs Usage)

**Figure 2:**
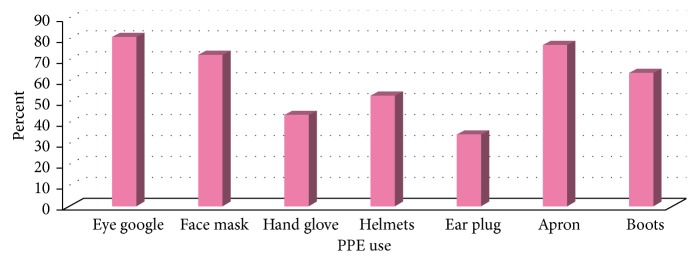
Status of PPE uses of respondents, among Axum and Adwa welding workers, September 2013.

From the total respondents, 225 (86.5%) workers used personal protective equipments. Among respondents who used personal protective equipments, eye goggle accounts the highest percent (80.8%), followed by an apron (76.9%), and the least (34.2%) wore ear plug (Figure 2).

Most of the respondents 200 (76.2%) used PPE because they knew the advantage of using personal protective equipments, but very small proportions of the respondents were using PPE by observing others as well as forced to use 17 (6.5%) and 6 (2.3%), respectively. The reasons given by 35 (15.5%) respondents of not using PPE were absence of PPEs by 41.3%, lack of knowledge about PPE among 23.9%, decrease in work performance in 32.6%, and uncomfortable feeling during use by 2.2% workers.

From the total respondents, 205 (78.8%) answered that they had work regulations in their respective working area even if more than half of the respondents 111 (54.2%) answered that these work regulations were not documented. Similarly, 205 (78.8%) respondents answered that they had working guideline in their respective working environment. Thirty-one (15.1%) of the respondents did not follow the working guideline listed in their working place. Out of the total study subject, 208 (80.0%) workers mentioned that work-related orientation was given when they were recruited to work.

### 3.4. Associated Factors for Knowledge Level and Safe Practices of Workers

Sociodemographic variables such as age, educational level, work experience, work type, and environment-related variables such as working safety training, work regulation, and working guidelines were assessed to see the association between them and the knowledge to occupational hazards among welding workers. The Odds ratio (OR) was calculated for the variables to compare the relative knowledge levels of those who had the information with that of those who had not. Logistic regression analysis by backward method was used to assess the relative effect of independent variables (sociodemographic and environmental characteristics) on the dependent variables (knowledge level and safe practice). All variables were taken to multivariate analysis.

Variables such as work experience, work type, safety training, and work regulation guideline had significant association with the knowledge of respondents. Workers who had more than five years of work experience are more than 56% less likely to be not knowledgeable as compared to workers who had less than one-year work experience, AOR: 0.44 (0.19, 0.99). Workers whose their work type is metal work are 62% less likely to be not knowledgeable as compared to workers whose work type is wood work, AOR: 0.38 (0.22, 0.65). Similarly, respondents who got safety training are 67% less likely to be not knowledgeable as compared to workers without safety training, AOR: 0.33 (0.17, 0.63). Welding workers with work regulation are 69% less likely not to have knowledge on occupational hazards as compared to workers without work regulation in their working environment, AOR: 0.31 (0.15, 0.67) (Table 3).

### 3.5. Safe Practice of Workers

Sociodemographic variables such as age, educational level, work experience, work type, and environment-related variables such as working safety training, work regulation, and working guidelines were assessed to see the association between them and the knowledge to occupational hazards among welding workers. The Odds ratio (OR) was calculated for the variables to compare the relative knowledge levels of those who had the information with that of those who had not. Logistic regression analysis by backward method was used to assess the relative effect of independent variables (sociodemographic and environmental characteristics) on the dependent variables (knowledge level and safe practice). All variables were taken to multivariate analysis. In this section, educational status, work experience, safety training, and availability of work regulation were found to be associated with PPE use. Workers with certificate and above level of education are more than 13 times more likely to use personal protective equipments than workers with primary school AOR: 13.20 (10.65, 16.46). Workers with safety training were 98% more likely to use personal protective equipments as compared to workers without safety training AOR: 0.02 (0.01, 0.09) (Table 4).

## 4. Discussions

The study aimed to determine workers level of awareness towards occupational hazards and their adherence to safety measures in Axum and Adwa. The mean score of respondents to the knowledge questions out of ten questions was 4. Respondents' score of 4 and above was considered knowledgeable on occupational hazards. In this study, the overall knowledge of respondents to occupational hazards was 44.2% which is lower than that in a study conducted in Addis Ababa where 69.5% respondents knew the presence of different kinds of occupational health and safety information and a study conducted in Adwa Textile factory on safety information where 68.7% respondents knew the presence of different kinds of occupational health and safety information [10, 11]. This might be because most workers were nonwelding-related professionals. Most workers had good knowledge on electrical hazards, 202 (77.7%), and accident prevention, 220 (84.6%), when compared to the other knowledge questions.

The study had revealed that 67.7% of respondents had knowledge about personal protective equipments; nearly similar result was reported from study conducted in India among garment workers, 75.2%, and Adwa textile factory workers, 72.3% [11, 12]. As one component of safe practices, 86.5% of study subjects used personal protective equipments. This is higher compared with the other components of safe practices in which 56.1% washed hands before eating, 75.6% used gloves, and 85.0% covered lids of containers after use [13]; this might be because we did not consider the frequency of using personal protective equipments in interviewing respondents. Among respondents who used personal protective equipments, eye goggle accounts the highest percent, 80.8%, followed by an apron, 76.9%, and the least (34.2%) wore ear plug.

In this study, work experience had significant association with the knowledge level of respondents that workers who had more than five years work experience are more than 56% less likely to be not knowledgeable (56% more likely to be knowledgeable) as compared to workers who had less than one-year work experience AOR: 0.44 (0.19, 0.99) similar with study conducted in Addis Ababa and Adwa town [10, 11].

Our study has revealed that health and safety training had significant association with knowledge level of respondents (AOR: 0.33 (0.17, 0.63)). This was supported by a study carried out in China in which the largest differences of mean score were found in the factors of safety training between large enterprises and small enterprises in creating safety climate. And a literature review on assessing occupational safety and health training suggested that reports were found and gave overwhelming evidence to show the merits of training in increasing workers' knowledge of job hazards and in effecting safer work practices and other positive actions in a wide array of worksites. Reports from selected surveys and investigations of worker injuries and workplace fatalities were also accessed with many implicating lack of training as a contributing factor to the mishaps. Still other studies, workplace training devoted to first aid instruction showed linkage to reduced worker injury rates, suggest that even this kind of training has benefits to job safety overall [14, 15].

The use of training and supervision during hazardous tasks has increased safety knowledge. In contrast to another study conducted in Addis Ababa, there was no significant association between health and safety training and safety information knowledge level [10, 16]. Although there were no similar studies for comparison, presence of work regulation had positive association on level of knowledge towards occupational hazards (AOR: 0.31 (0.15, 0.67)).

In our study, educational status had significant association with personal protective equipment usage in which workers with certificate and above level of education are more than 13 times more likely to use personal protective equipments than workers with primary school (AOR: 13.20 (10.65, 16.46)). It is coherent with different researches in such a way that lack of adherence to personal protective equipments is probably due to individual factor (educational level and work experience), and the low level of education may have also contributed to the inadequate knowledge and the nonuse of personal protective measures. This was also true with the research conducted in Addis Ababa in which educational level was statistically significant with glove and boot usage (AOR: 1.90 (1.05, 3.47)) and (AOR: 3.29 (1.72, 6.28)), respectively [10, 17, 18].

Similar with knowledge level of respondents, safety training also had significant association with personal protective equipment usage (AOR: 0.02 (0.01, 0.09)). It is similar from a study conducted in Hong Cong, China, that being informed of safety precautions by health and safety training and being supplied with safety information by supervisor were the significant factors leading to safe practice, and a study conducted among Australian grain factory workers, as well as a study conducted in Adwa town among textile workers, showed that PPE use was found to be associated with farm chemical training, the authors suggest that training is likely to be an important intervention for reducing farmers' pesticide exposure [10, 13, 19].

This study had a moderate larger sample size and interview type of data collection technique that enabled to have accurate data, and analyze in a quantitative manner the interrelationship among the knowledge and practice related to safety information as well as exploring factors that affect these outcomes. Some possibility of limitation of this study probably. Social desirability bias and self-reports (subjects might give socially acceptable responses), and all components of safe practices were not assessed.

Generally, nearly half of respondents had knowledge about occupational hazards, high proportion of study subjects used personal protective equipment, work experience, and safety training as the main sources of knowledge. Workers who had more than five years work experience and got training were found to have more knowledge. Safety training is the common determinant factor to increase knowledge level and safe practice of workers. Being certified and above educational status and having work regulation in their workplace were found to be more PPE users. As safety training was the common factor to increase for both knowledge level and safe practices, employers and trainers should work on workers to be accessed with training. Workers should be from welding-related professionals or should be given an orientation related to welding and its risk factors. Further research should be done to assess knowledge and practice on the health and safety outcome and other components of safe practices.

## Figures and Tables

**Table 1:** Distribution of sociodemographic characteristics of the respondents, Axum and Adwa welding workers, September 2013 (*n*=260).

Variable	Number	Percent
Age		
Less than 20 years	62	23.8
20–24 years	63	24.2
25–29 years	87	33.5
Greater than 29 years	48	18.5

Educational status		
Primary school	49	18.8
Secondary school	183	70.4
Certificate and above	28	10.8

Work type		
Metal work	129	49.6
Wood work	131	50.4

Profession		
Welding related	7	2.7
Nonrelated	253	97.3

Work experience		
<1 year	38	14.6
1–5 years	110	42.3
>5 years	112	43.1

**Table 2:** Knowledge level of respondents on occupational hazards among Axum and Adwa welding workers, September 2013 (*n*=260).

Knowledge variables	Number	Percent
Ever heard about occupational hazards		
Yes	135	51.9
No	125	48.1

Knowledge to fire hazards		
Yes	170	65.4
No	90	34.6

Knowledge to electrical hazards		
Yes	202	77.7
No	58	22.3

Knowledge to chemical hazards		
Yes	153	58.8
No	107	41.2

Knowledge to biological hazards		
Yes	122	46.9
No	138	53.1

Knowledge to ergonomically hazards		
Yes	118	45.4
No	142	54.6

Knowledge to mechanical hazards		
Yes	150	57.7
No	110	42.3

Knowledge to psychosocial hazards		
Yes	129	49.6
No	131	50.4

Knowledge to radiation hazards		
Yes	122	46.9
No	138	53.1

Knowledge of accident prevention		
Yes	220	84.6
No	40	15.4

Knowledge of safety measures		
Yes	176	67.7
No	84	32.3

Knowledge of occupational hazards		
Knowledgeable	115	44.2
Less knowledgeable	145	55.8

**Table 3:** Logistic regression analyses of the relative effect of sociodemographic and environmental variables on knowledge level, among Axum and Adwa welding workers, September 2013 (*n*=260).

Variables	Knowledge (≥mean score and <mean score)			
	Yes^a^	No	Crude OR (95% CI)	Adjusted OR (95% CI)
	*n* (%)	*n* (%)		
Age				
Less than 20 years	42 (67.7)	20 (32.3)	1.00	
20–24 years	41 (65.1)	22 (34.9)	0.89 (0.42, 1.87)	
25–29 years	39 (44.8)	48 (55.2)	0.39 (0.19, 0.76)^*∗∗*^	
Greater than 29 years	23 (47.9)	25 (52.1)	0.44 (0.20, 0.95)^*∗*^	

Level of education				
Primary school	31 (63.3)	18 (36.7)	1.00	
Secondary school	100 (54.6)	83 (45.4)	0.70 (0.36, 1.34)	
Certificate and above	14 (50.0)	14 (50.0)	0.58 (0.23, 1.49)	

Work experiences				
<1 year	25 (65.8)	13 (34.2)	1.00	
1–5 years	70 (63.6)	40 (36.4)	0.91 (0.42, 1.96)	
>5 years	50 (44.6)	62 (55.6)	0.42 (0.19, 0.90)^*∗*^	0.44 (0.19, 0.99)^*∗*^

Work type				
Wood work	92 (70.2)	39 (29.8)	1.00	1
Metal work	53 (41.1)	76 (58.9)	0.029 (0.18, 0.49)^*∗∗∗*^	0.38 (0.22, 0.65)^*∗∗∗*^

Safety training				
No	123 (62.4)	76 (37.6)	1.00	1
Yes	22 (35.5)	40 (64.5)	0.34 (0.19, 0.61)^*∗∗∗*^	0.33 (0.17, 0.63)^*∗∗∗*^

Availability of work regulation				
No	41 (74.5)	14 (25.5)	1.00	1
Yes	104 (50.7)	101 (49.3)	0.35 (0.18, 0.68)^*∗∗*^	0.31 (0.15, 0.67)^*∗∗*^

Availability of work guideline				
No	38 (69.1)	17 (30.9)	1.00	
Yes	107 (52.5)	97 (47.5)	0.49 (0.26, 0.93)^*∗*^	

^*∗∗∗*^Significant at *p* value <0.001; ^*∗∗*^significant at *p* value <0.01; ^*∗*^significant at *p* value <0.05. Yes^a^ = knowledgeable; no = less knowledgeable.

**Table 4:** Logistic regression analyses of the relative effect of sociodemographic and environmental variables on PPE use, among Axum and Adwa welding workers, September 2013 (*n*=260).

Variables	PPE use			
	Yes^a^	No	Crude OR (95% CI)	Adjusted OR (95% CI)
	*n* (%)	*n* (%)		
Age				
Less than 20 years	55 (88.7)	7 (11.3)	1.00	1.00
20–24 years	45 (71.4)	18 (28.6)	3.14 (1.21, 8.19)^*∗*^	
25–29 years	78 (89.7)	9 (10.3)	0.91 (0.32, 2.58)	
Greater than 29 years	47 (97.9)	1 (2.1)	0.17 (0.02, 1.41)	

Level of education				
Primary school	43 (87.8)	6 (12.2)	1.00	1.00
Secondary school	162 (88.5)	21 (11.5)	0.35 (0.11, 1.14)	2.60 (0.67, 10.01)
Certificate and above	20 (71.4)	8 (28.9)	0.32 (0.13, 0.83)^*∗*^	13.20 (10.65, 16.46)^*∗∗∗*^

Work experiences				
<1 year	27 (71.1)	11 (28.9)	1.00	1.00
1–5 years	94 (85.5)	16 (14.5)	0.42 (0.17, 1.01)	0.24 (0.06, 0.91)
>5 years	104 (92.9)	8 (7.1)	0.19 (0.07, 0.52)^*∗∗*^	0.03 (0.003, 0.34)^*∗∗*^

Safety training				
No	164 (82.8)	34 (17.2)	1.00	1.00
Yes	61 (98.4)	1 (1.6)	0.08 (0.01, 0.59)	0.02 (0.01, 0.09)^*∗∗*^

Availability of work regulation				
No	35 (63.6)	20 (36.4)	1.00	1.00
Yes	190 (92.7)	15 (7.3)	0.12 (0.65, 0.30)^*∗∗∗*^	0.06 (0.02, 0.21)^*∗∗∗*^

Availability of work guideline				
No	40 (72.7)	15 (27.3)	1.00	
Yes	184 (90.2)	20 (9.8)	0.29 (0.14, 0.62)^*∗∗*^	

^*∗∗∗*^Significant at *p* value <0.001; ^*∗∗*^significant at *p* value <0.01; ^*∗*^significant at *p* value <0.05. Yes^a^ = knowledgeable; no = less knowledgeable.

## Data Availability

Data and other required materials can be submitted if requested.
